# Dietary Melatonin Supplementation Could Be a Promising Preventing/Therapeutic Approach for a Variety of Liver Diseases

**DOI:** 10.3390/nu10091135

**Published:** 2018-08-21

**Authors:** Francesca Bonomini, Elisa Borsani, Gaia Favero, Luigi F. Rodella, Rita Rezzani

**Affiliations:** 1 Anatomy and Physiopathology Division, Department of Clinical and Experimental Sciences, University of Brescia, Viale Europa 11, 25123 Brescia, Italy; elisa.borsani@unibs.it (E.B.); gaia.favero@unibs.it (G.F.); luigi.rodella@unibs.it (L.F.R.); rita.rezzani@unibs.it (R.R.); 2 Interdipartimental University Center of Research “Adaption and Regeneration of Tissues and Organs—(ARTO)”, University of Brescia, 25123 Brescia, Italy

**Keywords:** melatonin, phytomelatonin, diet, oxidative stress, liver, mitochondria

## Abstract

In the therapeutic strategies, the role of diet is a well-established factor that can also have an important role in liver diseases. Melatonin, identified in animals, has many antioxidant properties and it was after discovered also in plants, named phytomelatonin. These substances have a positive effect during aging and in pathological conditions too. In particular, it is important to underline that the amount of melatonin produced by pineal gland in human decreases during lifetime and its reduction in blood could be related to pathological conditions in which mitochondria and oxidative stress play a pivotal role. Moreover, it has been indicated that melatonin/phytomelatonin containing foods may provide dietary melatonin, so their ingestion through balanced diets could be sufficient to confer health benefits. In this review, the classification of liver diseases and an overview of the most important aspects of melatonin/phytomelatonin, concerning the differences among their synthesis, their presence in foods and their role in health and diseases, are summarized. The findings suggest that melatonin/phytomelatonin supplementation with diet should be considered important in preventing different disease settings, in particular in liver. Currently, more studies are needed to strengthen the potential beneficial effects of melatonin/phytomelatonin in liver diseases and to better clarify the molecular mechanisms of action.

## 1. Introduction

Chronic liver diseases represent an important and underestimated world public health problem. At present, worldwide estimations show that 844 million people have chronic liver diseases, with a mortality rate of 2 million deaths per year [1]. For comparison, another public well recognized health disease is diabetes afflicting 422 million with 1.6 million deaths [2]. However, the majority of chronic liver diseases can be cured (chronic hepatitis C) and prevented or treated (chronic hepatitis B) and so essentially ignored as public health problems [3]. The aetiology of liver failure is unidentified but liver inflammation represents the main mechanism for the progression of chronic liver diseases, whatever the cause [4]. Inflammation causes hepatocyte necrosis and induces the progression of fibrosis to cirrhosis then hepatocellular carcinoma [5]. During this pathological process, chronic oxidative stress, with the formation of free radicals and reactive oxygen species (ROS), plays an important role and the mitochondria seem to be pivotal. Indeed, mitochondrial ROS stabilize the hypoxia-inducible factor in the cytosol and, thus, trigger the cellular adaptation during hypoxia [6,7,8]. However, the excessive production of ROS under various pathological conditions can damage the mitochondrial proteins, lipids and DNA [9].

New frontiers for planning more effective therapies should consider oxidative stress and mitochondria as targets. At this regard, the Mediterranean or other diets, rich in fruits and vegetables, seem to have an important role in prevent or treat several diseases. The healthy effect of these diets is linked to benefits of eating fruits and vegetables for the presence in these products of antioxidants, like vitamins C and E, flavonoids and other molecules [10]. Among them, melatonin was identified in plants, medical herbs and many foods, like meat, milk, rice, tomato, banana, potato and in several beverages, that is beer, pomegranate juice and red wine [11,12]. In particular, the melatonin of plant origin has been properly named phytomelatonin. Moreover, melatonin, which is considered as indoleamine having a fundamental role in the neuroimmunoendocrine system regulation [13,14], is also a potent antioxidant scavenging hydroxyl free radicals and related reactants [15,16]. Furthermore, it promotes antioxidant enzyme production [17] and protects mitochondrial activity also from ROS insult [18].

In the following paragraphs, the classification of liver diseases, considering the involvement of mitochondria and oxidative stress and the beneficial effects of melatonin/phytomelatonin supplementation will be examined. It is discussed the improvement of circulating melatonin through melatonin/phytomelatonin rich foods ingestion as a potential adjuvant therapy. To date, few data are available regarding the effect of dietary melatonin/phytomelatonin supplementation in different diseases and information regarding its possible use in liver diseases are scarce. Nevertheless, they are enough to suppose its beneficial effect in hepatic pathologies considering the well-established antioxidant and anti-inflammatory properties of melatonin/phytomelatonin.

## 2. Liver Diseases

Liver diseases could be summarized in two big categories: neoplastic liver diseases (hepatocellular carcinoma, hepatoblastoma, primary hepatic angiosarcoma, cholangiocarcinoma) and non-neoplastic liver diseases. In this last category, the following principal subcategories can be identified as: (1) alcoholic liver disease (ALD); (2) non-alcoholic liver disease, such as non-alcoholic fatty liver diseases (NAFLD), non-alcoholic steatohepatitis (NASH); (3) hepatic cholestasis; (4) human hepatitis viruses; (5) hepatic toxicity.

### 2.1. Neoplastic Liver Diseases

Liver tumours are not the most frequent but represent the principal complication of chronic liver diseases or cirrhosis related to hepatitis B or C virus infection [19,20]. So, research in this field is very promising and significant advances have been reached in diagnosis and treatment [21]. The most common tumour is the hepatocellular carcinoma. It is the third leading cause of cancer death worldwide and is therefore recognized as a serious disease [22]. Two tumours less widespread are the hepatoblastoma, a rare childhood cancer [23,24] and the primary hepatic angiosarcoma of vascular endothelial cell origin, which represents only 1.8% of all hepatic diseases [25]. Finally, cholangiocarcinoma affects the biliary tract epithelium with an increased incidence in recent years [26].

### 2.2. Non-Neoplastic Liver Diseases

Alcoholic liver disease (ALD) is due to an excessive alcohol consumption representing an important cause of mortality. Its risk is related to different factors, such the intensity and duration of alcohol intake and genetic [27]. Steatosis, that is the excessive accumulation of triglycerides in the hepatocytes, practically is present in all cases. More than one in five patients develop alcoholic hepatitis and one-third of these will suffer cirrhosis, which may develop to hepatocellular carcinoma. In particular, alcohol compromised the intestinal barrier inducing alcoholic hepatitis due to an increased presence of bacteria-derived lipopolysaccharide (LPS) in the portal blood, this latter produces an altered inflammatory response, resulting in tissue injury [28,29].

Non-alcoholic fatty liver disease (NAFLD) is a condition characterized by no excessive alcohol intake, or no alcohol assumption at all (<20 g/day in females and <40 g/day in males) [30,31]. One-third of adults in Western countries are affected by this pathology. In this category, a large number of diseases has been identified, such as hepatic steatosis, non-alcoholic steatohepatitis (NASH) and cirrhosis. Moreover, some medical conditions are frequently associated, such as diabetes, obesity and metabolic syndrome [32].

Hepatic cholestasis is a condition where the bile flow from the liver to the intestine is blocked with the consequent accumulation of hydrophobic bile acids in the liver and plasma. Hepatic cholestasis is provoked by diverse inducing factors, including drug treatment, bile duct ligation, inherited and syndromic forms (e.g., disorders of primary bile acid synthesis, abnormalities in hepatocyte transport of bile constituents, intrahepatic biliary hypoplasia) [33,34].

Among the five human hepatitis viruses (A–E), hepatitis B virus (HBV), hepatitis C virus (HCV) and hepatitis delta virus (HDV) may induce either transient or chronic disease. They are the most dangerous for the liver because they are responsible of several liver failure conditions, such as fulminant hepatitis, cirrhosis and hepatocellular carcinoma [35].

Hepatic toxicity can be induced by a large number of drugs and the risk increases when the liver is highly involved in metabolizing the drug or the dose is higher than 50 mg/day. Mitochondrial dysfunction and lipid dysmetabolism are the causes of liver failure due by the drug itself and/or by the reactive metabolites generated. Other type of hepatic toxicity can be induced by iron overload or carbon tetrachloride (CCl4) exposure. Iron, an essential constituent of the body, can be excessively present due to a wide range of acquired and hereditary conditions, such as the hereditary hemochromatosis [36]. This iron overload leads to various toxic effects, among them the most common is liver damage, giving rise to fibrosis and cirrhosis [37]. Moreover, the major outcome of CCl4 exposure is hepatotoxicity, detectable by clinical signs, biochemical alterations or histological examination [38].

## 3. Role of Mitochondrial Dysfunction in Liver Diseases

Mitochondria, the powerhouse of the cells, are dynamic organelles and participate in many other cellular functions and pathways, in particular they are considered the major source of intracellular ROS production [39,40]. Moreover, a number of antioxidant enzymes such as catalase (CAT), superoxide dismutase (SOD) and glutathione peroxidase maintain the ROS level within the physiological range [41]. Oxidative stress occurs when the free radicals’ production exceeds the activity of the antioxidant defence system and alters the chemical structure of any cell component. Thus, an imbalance in the cellular and/or extracellular concentration of free radicals contributes significantly to organ injury [42]. In fact, the excessive formation of ROS and reactive nitrogen species (RNS), glutathione depletion and protein alkylation are major events associated with mitochondrial dysfunction and represent critical initiating events in most forms of chronic liver diseases [43,44,45,46,47] and in particular tumours [48]. So, mitochondrial membrane components, such as lipid and protein and DNA are modified by the oxidative action of ROS [49]. They represent the key factors for liver damage and induce a variety of conditions compromising liver function, including ischemia-reperfusion injury (I/R) [50].

Another important step is the impairment of lipid metabolism by the presence of lipid peroxyl radical (PLOO·) [51,52], that is produced during lipid peroxidation and able to propagate the chain reactions and peroxynitrite (ONOO^−^), which is considered a dynamic initiator of lipid breakdown. In addition, the role of Sirtuin 1 (SIRT1)—a protein deacetylase—has been also studied as a regulator of mitochondrial dynamics. It has been found that SIRT1 was depleted by free fatty acids and, in turn, SIRT1-dependent deacetylation of mitofusin 2, a protein critical for maintaining mitochondrial integrity and functioning. [53].

Moreover, it has been showed that levels of microsomal membrane rigidity, hepatic malondialdehyde (MDA), nitric oxide (NO) and 8-hydroxydeoxyguanosine (8-OH-dG), the commonly used marker of oxidative stress-derived DNA damage, were altered in hepatic pathologies [54,55,56]. In addition, NO is produced by several forms of NOS (nitric oxide synthase), also in the mitochondria named constitutive (c-mtNOS) and inducible (i-mtNOS) [57]. NO actively interacts with components of the respiratory chain in particular cytochrome c oxidase and interferes with respiratory chain complexes [58,59,60].

Another molecule involved in oxidative stress-induced mitochondrial alterations is cardiolipin. Its oxidation results in both decreased activity of respiratory chain complexes [61] and apoptosis induction [62]. A number of oxidative phosphorylation proteins, such as complex I–IV, for their activities need cardiolipin and interact with it [63,64,65,66]. Moreover, excessive free radical generation in mitochondria can also triggers a condition called mitochondrial permeability transition, MPT, in which several proteins of the inner mitochondrial membrane, form a supramolecular structure which acts as a non-specific pore [67] causing dissipation of the mitochondrial membrane potential and loss of ATP synthesis capacity. The cardiolipin oxidation caused MPT initiation [68] and the release of cytochrome c from the mitochondria into cytosol, which is involved in the early events of apoptosis [69,70].

Several data showed that oxidative stress upregulated NF-kB, which is considered a pro-inflammatory protein able to induce cytokines production, among which interleukin 1β, interleukin-6 and TNF-α [71,72,73]. It has been shown that in CCl4 hepatotoxicity there was an increase of TNF-α and also overexpression of a type I membrane receptor of the TNF-receptor superfamily named Fas, which plays a role in macrophages pathogenesis [74,75,76]. Moreover, liver disease was related with increased serum aspartate aminotransferase (AST) and alanine aminotransferase (ALT) levels, which indicated considerable hepatocellular injury [76].

Mitochondria are key organelles also involved in apoptosis, proliferation and calcium homeostasis. The consequence of their impairment may lead to tumour [48]. Recent reports strongly support a link between mitochondria and cancer. ROS-induced activation signalling cascades, mainly the Ras/Raf via oxidative modification and activation of mitogen-activated protein kinases (MAPK) [77] has been described as responsible of liver cancer progression [78] but other molecules seem to be implicated such as NF-kB [79] and some cytokines [80]. Moreover, ROS directly alter cell signalling via many pathways, the most important of which is the oxidation of thiol groups in several key proteins involved in apoptosis and cell cycle regulation [81,82,83]. Mitochondrial proteins are encoded by the nuclear genome but mitochondria act also on several nuclear functions by a retrograde control through a variety of different mechanisms [84], leading also to tumour [85,86,87].

In conclusion, liver inflammation, fibrosis and tumours are evidently connected with chronic oxidative stress. Indeed, oxidative stress promotes parenchymal damage and hepatocyte loss with the consequent recruitment of immune effectors at the injured site [48,88,89,90].

## 4. Melatonin and Potential Nutritional Impact in Health and Diseases

To date, therapy for liver diseases is evolving with a substantial number of trials of promising new agents [91] based on the fundamental pathophysiological mechanisms which centres on ROS production and mitochondria alterations. These are antioxidant strategies, which include supplementation of vitamins E and D, Mediterranean and other diets [92] and bioactive components from foods among which melatonin [93].

Melatonin, *N*-acetyl-5-methoxytryptamine, is a highly conserved indoleamine molecule found in all microorganisms, plants and animals [94,95,96]. Moreover, it is considered a safe molecule, in fact animal and human studies reported that short-term use of melatonin does not cause adverse effects, even in extreme doses. Only mild side effects, such as dizziness, headache, nausea and sleepiness have been reported. Likewise, clinical studies indicate that long-term melatonin treatment causes the same adverse effects comparable to the placebo [97].

This indoleamine was first discovered in the bovine pineal gland [98]. Successively, it was identified also in plants in 1995 and named phytomelatonin in 2004 [99,100,101,102]. The discover improved the interest in investigating the content and the potential beneficial effects of phytomelatonin in plants, in particular in edible plants. 

It is important to underline that melatonin secretion and/or serum levels change through a human lifetime. It is noteworthy that after childhood and in particular in advanced age, melatonin secretion decreases and it may be related to pathological conditions [103,104,105], so strategies to raise melatonin levels, such as increase dietary consumption, become important especially in elderly [11].

In the following paragraphs an overview of the most important aspects of melatonin and of phytomelatonin, describing the differences among their synthesis, their presence in foods and their role in nutrition, is presented. Finally, the positive effects of both substances in physiological and pathological conditions are discussed.

### 4.1. Melatonin and Phytomelatonin

Melatonin and phytomelatonin are structurally the same molecule [106], but, as previously reported, melatonin refers to animal origin and the term phytomelatonin to plant origin. In particular, the term phytomelatonin was introduced for the first time in 2004 by Blask et al. [101].

#### 4.1.1. Melatonin Synthesis 

Melatonin is secreted mainly by the pineal gland at night under normal light/dark conditions. The rhythm of endogenous secretion is generated by the suprachiasmatic nuclei and entrained to the light/dark cycle. In particular, there is an increase of biosynthesis during the night (from 6:00 p.m. to 5:00 a.m.) and a peak at midnight [107].

**Figure 1:**
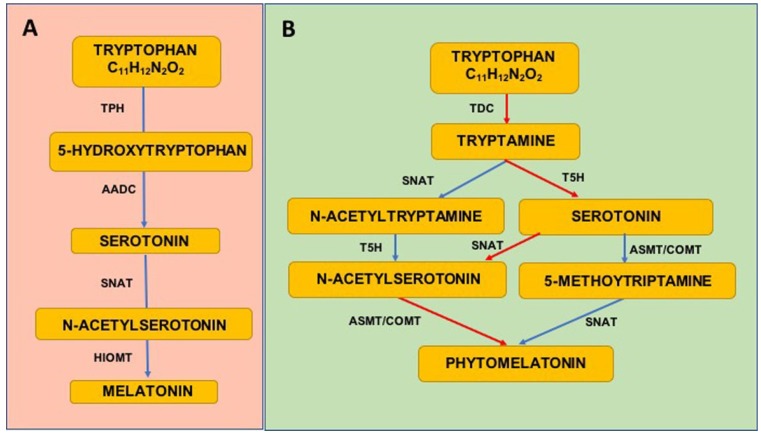
Biosynthetic pathways of melatonin in animals (**A**) and in plants (**B**). Red arrows indicate the most relevant way of phytomelatonin synthesis.

Briefly, melatonin is synthesized in pineal gland through a four-step pathway from its precursor the essential amino acid tryptophan taken up from the circulation. The amount of melatonin formation is due to four enzymes activities, the first two, tryptophan hydroxylase (TPH) and aromatic amino acid decarboxylase (AADC), convert tryptophan in serotonin. After these steps, serotonin is converted in melatonin trough other two process mediated by the activity of other two enzymes, serotonin-*N*-acetyl transferase (SNAT) and hydroxyindole-*O*-methyl transferase (HIOMT) [106] (Figure 1A).

#### 4.1.2. Phytomelatonin Synthesis 

Phytomelatonin has a different biosynthetic pathway respect to melatonin. In particular, it has more alternative routes of synthesis reflecting a greater capacity to adapt to metabolic changes. 

Briefly, as follow, three different principal ways have been described. Tryptophan is converted into tryptamine by the enzyme tryptophan decarboxylase (TDC) and then Tryptamine, by tryptamine 5-hydroxylase (T5H), is catalysed to serotonin. Serotonin via *N*-acetylation, mediated by the enzyme serotonin *N*-acetyltransferase (SNAT), is converted in *N*-acetylserotonin that is methylated by acetylserotonin methyl transferase (ASMT), which generates phytomelatonin. Another enzyme, caffeic acid *O*-methyltransferase (COMT), could also mediate methylation of *N*-acetylserotonin in plants [108]. Serotonin may also be converted into 5-methoxytryptamine by ASMT or COMT and finally generate phytomelatonin via SNAT activity. Moreover, phytomelatonin could be generated from tryptamine by formation of *N*-acetyltryptamine by a pathway mediated by SNAT. *N*-acetyltryptamine via T5H activity is converted into *N*-acetylserotonin and then using the previous described pathway in melatonin. Finally, phytomelatonin can be generated through the formation of 5-methoxytryptamine [106,109,110] (Figure 1B). 

#### 4.1.3. Melatonin in Diet 

Currently, there are few data in literature regarding the melatonin concentration in foods of animal origin [11,111].

Tan et al. [111] reported, for the first time, melatonin’s presence in dietary meats such as chicken, lamb, beef, pork but also in fish, eggs and colostrum (Table 1). The melatonin levels in meats were similar to concentration observed in foods of vegetal origins and are in the range of ng/g. It is interestingly to note that chicken skin and skeletal muscle contain more melatonin than heart and liver [111]. The highest levels of melatonin in animal foods were found in eggs and fish. In particular, in solid dried eggs the melatonin content is 6.1 ± 0.95 ng/g, while in salmon is 3.7 ± 0.21 ng/g.

It was also found in animal milk and in mother milk consumed by the human beings [111,112]. In particular, the concentration of melatonin in milk is elevated during the night while it is reduced during the day, with a trend similar to that observed in blood. So, drinking milk produced during the night has a beneficial melatonin-dependent effect greater than daytime milk [113]. Moreover, melatonin levels were showed in powdered bovine colostrum, this content was higher than physiological blood levels in mammals but in fresh bovine colostrum it was lower and similar to the range of normal physiological plasma levels [114], so the contribution of melatonin from colostrum to the circulating melatonin levels in nursing new-borns could be significant as the melatonin synthesis by the pineal gland in the first weeks of life is not fully understood [115].

On the basis of these data, it is possible to suggest that that consumption of melatonin-rich foodstuff, presumably, increases the circulating melatonin levels in humans (Table 1).

#### 4.1.4. Phytomelatonin in Diet

It is widely recognized the presence of phytomelatonin in a variety of plants, including medicinal ones. In particular, melatonin has been described in fruits, vegetables, leaves and seeds. In crop plants, the highest values of phytomelatonin have been showed in the fruits of apple, cherry, tomatoes, peppers, grapes, coffee beans, mustard and almond seeds but it has also been quantified in cereals such as oat, corn, rice and barley [10,11].

Phytomelatonin levels described in plants, generally, range from picograms to micrograms per gram of tissue and multiple factors, both endogenous and exogenous—i.e., culture conditions and analytical assay type—may influence quantifications. 

Several studies have been conducted for phytomelatonin quantification in fruits and vegetables [10,96]. The phytomelatonin concentrations in cereals vary from several nanograms to several thousand picograms per gram [10,96]. In particular, in rice, the phytomelatonin contents are very different between pigmented and non-pigmented rice samples. The highest concentration of phytomelatonin in pigmented rice is 207.79 ± 3.18 ng/g (red rice) and this is followed by black rice (182.04 ± 2.79 ng/g) and black glutinous rice (73.81 ± 1.13 ng/g) [10,116]. In fruits the average amounts of melatonin are 0.47 ng/g in banana, 0.25 ng/g in tomato, 0.09 ng/g in cucumber, 0.009 ng/g in beetroot. In some fruits, the phytomelatonin concentration varies between cultivars, in grape skin the content of phytomelatonin range from 8.9 to 158.9 ng/g, in tart cherries 13.46 ± 1.10 ng/g and 11.26 ± 0.13 ng/g in strawberry [10,11,117]. It was also found at high levels in nuts and, recently, in kernels of four different pistachio varieties in which phytomelatonin reaches 230 μg/g [10,118]. Melatonin is also present in vegetables, in particular in tomatoes the concentration ranges from 4.11 to 114.52 ng/g (fresh weight) and has been quantified in different tomato varieties, the varieties Marbone and RAF presenting the highest melatonin concentrations (114.52 and 50.1 ng/g, respectively), whereas Lucinda and Catalina varieties show the lowest melatonin concentrations (4.45 and 4.11 ng/g, respectively) [10,119]. In red pepper fruits the phytomelatonin content ranged from 31.0 to 93.4 ng/g (dry weight) [10,120]. Mushrooms also contain melatonin at different concentrations ranging from 4300 to 13000 ng/g [11].

Moreover, very high phytomelatonin levels have been measured in some plants that are used as herbal medicines for centuries [121] in particular it was identified in the majority of these herbs, that is, phytomelatonin concentrations in *yinyanghuo* (*Epimedium brevicornum* Maxim) are of 1105 ng/g, in *sangbaipi* (*Mori Albae Cortex*) of 1110 ng/g and in *Sangye* (leaf of *Morus alba* L.) of 1510 ng/g [10,121]. So, the presence of phytomelatonin in beverages derived from plant products are not surprising. In fact, phytomelatonin has been quantified in large amount in the most popular beverages such as coffee, tea, juice, herbal infusions, wine and beer [11,96,122,123,124,125,126,127,128,129]. In alcoholic drinks like beer [122,130] and wine [127,131], the melatonin amount was of 0.09 ± 0.01 ng/mL in beer [130] and up to 129.5 ± 3.5 ng/mL in wine [122,130].

Considering that phytomelatonin originates from the main ingredients, also the fermentation process due to the yeast growth has been considered [122,132]. Melatonin synthesis by yeast during alcoholic fermentation has been observed and evaluated in white and red wines [132,133], beer [122], orange and pomegranate juices [10,134]. As for the other drinks, coffee usually contains high content of phytomelatonin because coffee beans contained phytomelatonin at a very high level and even much higher in roasted beans [125]. Phytomelatonin also existed in cacao [130] and balsamic vinegars [133] (Table 1).

In conclusion, the phytomelatonin presence in different foodstuff could be of interest for further study on the beneficial effects of a balanced diet in increasing circulating melatonin levels, also in pathological conditions.

### 4.2. Melatonin/Phytomelatonin and Health Effects

Melatonin/phytomelatonin beneficial effects for human health, especially, in retarding processes associated with aging and slowing of pathological conditions have been frequently discussed [135,136,137,138]. As previously reported, in recent years the nutritional importance of melatonin/phytomelatonin in foodstuffs has drawn increasing attention. In fact, it was showed that consumption of melatonin/phytomelatonin-containing foods increases circulating melatonin levels that are correlated with the total antioxidant potential in humans and animals [99,139].

It is estimated that, via the cascade reaction, one melatonin/phytomelatonin molecule potentially scavenges 10 free radicals, which contrasts with the classic antioxidants because they typically detoxify one radical per molecule [140]. Therefore, it has been deduced that the initial function of melatonin in organisms, in physiological conditions, was to serve as an antioxidant to scavenge a variety of ROS and reactive nitrogen species (RNS) and to protect plants from oxidative stress [15,99,140,141]. Exogenous application of melatonin/phytomelatonin increases antioxidant enzyme activities, while it decreases superoxide, hydrogen peroxide and malondialdehyde concentrations [142,143,144,145].

#### 4.2.1. Melatonin in Health

At present, the importance of circadian rhythmicity for human health and welfare has been widely demonstrated. Melatonin is recognized to alleviate feelings of jet lag, reducing of sleep onset latency and improving sleep quality [146] in dose between 0.5 and 5 mg.

The wide range of the reported properties of melatonin is related to its chronobiotic effect, such as cardioprotective, digestive or immunomodulator. Moreover, melatonin is involved in the regulation of mood and it is a scavenger of a number of ROS and RNS both in vitro and in vivo [147]. Many of its reported beneficial properties have been linked also with its antioxidant [16,148] and anti-inflammatory effects [111] which improve neurological function, retard processes linked to aging and slow the onset of different diseases such as neurodegenerative diseases, heart disease and diabetes [135,137,138,149].

The importance of melatonin in nutrition has become an element of increasing interest in the last years [111]. Melatonin can be ingested both as a normal part of the diet and from food supplements or functional foods, where melatonin is used as a bioactive ingredient [111]. In particular, melatonin can be found in meats (especially from chicken, beef, pork), fish and eggs, which represent the most used foodstuffs in developed countries. In addition, melatonin is present also in animal and plant products used in the preparation of animal food. The consequence of the consumption of melatonin-containing foods is the increase of circulating melatonin and so the antioxidant potential in humans and animals [99,139].

Melatonin is also implicated in lipid metabolism. In fact, in different species, the weight loss has been frequently associated with melatonin supplementation [150,151]. This can be due to the recruitment and activation of brown adipose tissue (BAT) by melatonin, which converts energy stored in fat into heat [150]. In an experimental study, genetically obese mouse supplemented with melatonin converted white adipose tissue to BAT by a process of de-differentiation and thereby reduced their white fat accumulation [152].

So, melatonin has a wide spectrum of action in human, improving the physiological conditions at different levels.

#### 4.2.2. Melatonin in Pathological Conditions

The disorders of circadian rhythmicity are characteristic of a variety of pathologies (e.g., neurological or psychiatric disorders, metabolic alterations) in humans. It is also known that disturbed circadian rhythmicity, whether due to living conditions (e.g., jet-lag) or natural circumstances (e.g., aging), can promote the development of specific pathologies, sleep disorders and metabolic syndromes (i.e., obesity, diabetes, hypocholesteraemia, cardiovascular diseases and cancer) [153,154,155]. Moreover, obesity induces changes in rhythms but the resulting internal desynchronization will, by itself, further aggravate obesity [156,157]. Many articles reported the anti-inflammatory effects of melatonin and linked these with an improvement in neurological function. The role of non-chronic inflammation in Multiple Sclerosis (MS) is well known. In fact, the initial pathogenesis of this disease has a strong inflammatory-demyelinating component [158]. Farhadi et al. [159] showed that, in addition to increasing the levels of pro inflammatory cytokines, patients with MS presented a decrease in serum levels of melatonin. In addition, Kang et al. [160] showed that supplementation with exogenous melatonin during the inflammatory-demyelinating process could be useful for the improvement of the myelin status of nerve fibres.

The nutritional impact of melatonin present in foodstuffs is probably underestimated. Animal studies have shown that melatonin supplementation improves the lipid metabolic profiles, lowers the blood levels of cholesterol and triglycerides and enhances the insulin sensitivity of many tissues in ob/ob fatty mice [161] and in diet-induced obese animals [162]. Clinical trials have confirmed the observations obtained from animal studies, demonstrating that melatonin supplementation also improves the metabolic profiles of the lipid and glucose in human subjects with diabetes or metabolic syndrome [149,163]. Additionally, it exhibited protective effects in fatty liver [164,165]. Other very recent reports underline the multiple potentialities of melatonin in different diseases (e.g., lupus nephritis, fibromyalgia, neuropathies) most of which related to its antioxidant and anti-inflammatory properties [166,167,168].

#### 4.2.3. Phytomelatonin in Health

Some studies suggested that intake phytomelatonin-rich foods may have health impacts via the increase of circulating melatonin [124,139,169,170,171].

The animals fed with walnuts showed an increase in concentrations of melatonin and a gain in total antioxidant capacity in blood, suggesting that walnuts in diet could provide beneficial effects as a food source of phytomelatonin [172]. Additionally, in a clinical trial with enrolment of young, middle-aged and elderly participants, the total antioxidant capacity after the intake of the experimental juice of the grape (*Vitis vinifera* cv. Tempranillo) increased significantly in all the groups [171,173], suggesting that the consumption of foods rich in phytomelatonin could be able to provide many health benefits [11]. Moreover, recently Sae-Teaw et al. [139] showed that the consumption of banana or fruit juices (orange and pineapple) significantly increased the serum melatonin concentration and especially at 120 min after the intake, in healthy male volunteers. This increase was related with a marked increase in antioxidant capacity of the serum, suggested by the significant increases in two indicators, that is, ferric reducing antioxidant power (FRAP) assay and oxygen radical antioxidant capacity (ORAC).

#### 4.2.4. Phytomelatonin in Pathological Conditions 

Few studies reported the effects of phytomelatonin in pathological conditions. Howatson et al. [174] showed that drinking cherry juice improved sleep quality of elderly individuals with insomnia supposing that sleep improvement is due to the exogenous phytomelatonin increase afforded by the cherry juice [12]. Other studies demonstrated the protective effects of coffee against liver diseases such as hepatic fibrosis, steatohepatitis and CCl4-induced liver cirrhosis in experimental animal models [175,176,177]. The hepatoprotective role of coffee could be attributed to the antioxidant properties of the coffee constituents and, in particular, to the high melatonin content in coffee [125]. Moreover, some reports have proven that melatonin effectively protects against liver pathologies [96,162,178,179,180,181]. Lamont et al. [182] showed that daily moderate consumption of red wine protected the heart against an experimental I/R injury and that the inhibition of melatonin receptors resulted in the attenuation of red wine-induced heathy effect. These results provided strong evidence that melatonin/phytomelatonin supplementation acts as a key player in cardioprotection. In a recent review, Jiki et al. [183] reported also that in a model of pulmonary hypertension, a chronic dietary melatonin/phytomelatonin treatment reduces right ventricle hypertrophy, improves ventricular function, reduces plasma oxidative stress and reduces cardiac interstitial fibrosis [183,184].

All these data support the beneficial effects of dietary melatonin/phytomelatonin intake in different diseases.

## 5. Melatonin/Phytomelatonin for Prevention of Oxidative Stress 

Considering the previous described presence of melatonin/phytomelatonin in food, plants and its possible use as a food addictive, their properties deserve attention [185].

Among the bioactivities that melatonin/phytomelatonin exhibits, there are a powerful antioxidant action and an involvement in modulation of lipids and carbohydrates metabolism [16]. Different studies demonstrated that melatonin has a key role in antioxidant defence acting directly on mitochondria, with a reduction of ROS generation and indirectly by endogenous antioxidant system stimulation [140,186,187,188,189,190,191,192]. Tan et al. [193,194] reported the first evidence of the melatonin antioxidant effect showing that this indoleamine is able to scavenge two molecules of hydroxyl radical and convert them to cyclic 3-hydroxymelatonin. Cyclic 3-hydroxymelatonin was found in the urine of human and animals under oxidative stress conditions and treated with melatonin [194]. Melatonin is also considered a powerful scavenger of RNS. As widely shown in different animal and vegetal models, melatonin protects against lipid peroxidation under various oxidizing conditions such as ionizing radiation, heavy metal toxicity and drug metabolism [195]. The exact mechanism by which melatonin and its products influence lipid peroxidation until now is not fully demonstrated. Melatonin has been shown to scavenge the lipid peroxyl radical (PLOO) [51,52]. Moreover, melatonin demonstrated to be more active respect to vitamin E in PLOO· neutralization and lipid peroxidation inhibition [51,196]. This indoleamine can neutralize ONOO^−^ and it is able to protect membrane lipids [197]. To note that melatonin is considered a lipid peroxidation inhibitor by its action in interfering with the radicals that trigger this process [198]. The amphiphilic properties and the small molecular size of this indoleamine help its diffusion into subcellular compartments. In vitro evidences demonstrated that melatonin can inhibit lipid peroxidation in brain homogenates, brain and liver microsomes and mitochondria [199]. The conservation of the integrity of the mitochondrial membrane reflects the action of this indoleamine in mitochondria-dependent apoptotic pathway modulation. So, the protective effects of melatonin on apoptotic mechanisms are well established [164]. Melatonin inhibits MPT pore by its protective action on a dimeric phospholipid found in the mitochondrial membrane called cardiolipin [190]. On the other hand, evidences have shown that melatonin could prevent oxidation of cardiolipin and attenuate apoptosis in pathological conditions and aging [190].

Mitochondrial dynamics are also modulated by melatonin. Melatonin induces mitochondrial fusion but it is able to reduce mitochondrial fission under toxic conditions [200,201,202,203]. In particular, melatonin acts on fusion/fission equilibrium by the modulation of oxidative stress or through mechanisms that are not fully understood. In this regard, Pei et al. [204] demonstrated that melatonin increased the expression of a protein mediator of mitochondrial fusion, mitofusin. New data also show that, when cellular damage has occurred, melatonin induces mitophagy, even if the mechanism is not fully understood and need more clarifications [187,205,206,207]. Recently reports suggested also that melatonin can act as an anti-cancer molecule via a pro-apoptotic effect in hepatocarcinoma cells [208]. Other data underline the fundamental opposite pro- and anti-apoptotic melatonin effects. In fact, it has also been suggested that melatonin exerts an anti-apoptotic role in toxic insults caused by anti-cancer chemicals like oxaliplatin or etoposide [209,210]. (Shokrzadeh M. et al., 2018, Waseem et al., 2017). In particular, pre-treatment with this indoleamine provides cell protection by modulating oxidative stress, mitochondrial functions and apoptosis-regulatory proteins. Nevertheless, it is important to note that melatonin reduces the effects of these chemotherapeutic agents significantly through the reduction of the level of DNA damage [210].

Some studies showed that melatonin, in liver pathophysiology, is able to influence various hepatic cell types, such as hepatocytes, hepatic stellate cells and cholangiocytes [205].

Melatonin supplementation has also been shown to scavenge directly free radicals in liver injury induced by ionizing radiations [211]. Moreover, 8-OH-dG levels were reduced after melatonin administration. Furthermore, MDA and NO levels were decreased with melatonin treatment, while the activity of endogenous antioxidant system was considerably improved [54,55,56]. Kang et al. [212] showed that melatonin protected the liver against I/R injury by heme oxigenase-1 overexpression. Moreover, melatonin downregulates autophagy related to the production of ROS during I/R through a mammalian target of the rapamycin-dependent mechanism [213,214].

Melatonin preserves mitochondrial physiology during oxidative stress. Melatonin may also modulate mitochondrial endogenous antioxidants via sirtuin 3 (SIRT3)/superoxide dismutase 2 (SOD2) signalling in the mitochondria to regulate the oxidative stress in this organelle [79,215]. The disruption of the mitochondrial respiratory cycle and ATP synthesis due to toxin exposure is restored by melatonin administration. In hepatic oxidative stress conditions, melatonin likely reduces electron leakage and free radical generation, thereby contributes to molecular damage protection at the mitochondrial level [151]. However, it has been showed that melatonin increases the expression of uncoupling protein (UCP) [216], a member of the mitochondrial anion carrier family, is considered to prevent mitochondrial superoxide generation and rendering electron flow through the respiratory complexes more efficient [217]. So, melatonin supplementation may decrease free radical generation in mitochondria in two different ways: by reducing electron leakage from the MPT pore and by promoting electron leakage via the uncoupling protein, which is the physiological pathway for decreasing free radical generation [218].

## 6. Melatonin Treatment in Liver Alterations

The most frequent alterations in liver disease are due to fibrosis, inflammation, steatosis and carcinogenesis. Liver fibrosis is considered a reversible process and regression of diseases at this stage is a key strategy in preventing the progression to chronic pathology. This fact has spurred efforts to uncover the mechanisms governing fibrosis for evaluating new therapeutic strategies [205]. Actually, the potential therapeutic approach for treatment of fibrosis is focused on deletion of the soluble factors released by hepatocytes, including inflammatory cytokines and ROS, which leading to the activation of hepatic stellate cells (HCSs) that are responsible of collagen secretion and fibrogenesis [219]. Moreover, in different experimental pathological conditions melatonin has been shown to decrease liver fibrosis [165,220,221].

Among the molecular mechanisms involved in fibrosis there was mitochondrial dysfunction that is characteristic of different liver diseases [44]. Different authors have reported that melatonin supplementation was able to improve hepatic mitochondrial function, reducing oxidative stress and increasing the activities of complexes I and IV of the mitochondrial respiratory chain [222,223,224,225,226]. Moreover, it was showed that melatonin strengthened the respiration rate and the increased acceptor control ratio value in mitochondria isolated from liver of an animal model of diabetes [227,228] inducing a reduction of liver damage [229]. Furthermore, in a recent work Kang et al. [205], evaluated the effect of melatonin against experimental liver fibrosis induced by CCl4 with particular attention on mitochondrial homeostasis. These authors showed that the treatment with melatonin prevented hepatic fibrosis by improving impairment of mitochondria biogenesis and mitophagy. Moreover, Das et al. [219], found that melatonin in hepatocytes had a protective role on mitochondrial dysfunction and it was able to inactivate fibrogenesis by HCSs inhibition. In particular, they showed that depletion of SIRT1 by free fatty acids was restored by melatonin and, in turn, SIRT1-dependent deacetylation of mitofusin 2, a protein critical for maintaining mitochondrial integrity and functioning. So, in this study melatonin protected mitochondrial function and morphology.

In a study by Lowes et al. [230] it was showed that antioxidants that act preferentially in mitochondria, among which melatonin, reduced mitochondrial damage and organ dysfunction and decreased inflammatory responses. Ebaid et al. [76] showed also that melatonin, especially if administered in combination with folic acid, may mediate oxidative stability and in turn NF-κB activation downregulation and the subsequent suppression of the inflammatory cascade activation. The effect of this combined treatment was also confirmed through histopathological evaluations [76].

Studies on the protective effect of melatonin by reducing oxidative stress induced by NAFLD and NASH are poor. Melatonin seems to act via restoring a proper balance in mitochondrial metabolism [231]. Das et al. showed that this indoleamine could restore the depressed proton leak of the mitochondria so decreasing oxidative damage resulted from greater metabolic flux [219]. Some studies considered the protective effects of melatonin against NAFLD in different animal models due to its antioxidative function [203,219,232]. Moreover, in other studies by Gonciarz et al. [233,234,235], patients with NASH administration of 10 mg/day melatonin for 4, 12 and 24 weeks resulted in a significant increase of plasma melatonin and beneficial effects on liver enzymes making it a suitable molecule for use in human liver diseases.

Finally, recent advances suggested that melatonin could exert a pro-apoptotic effect in hepatocarcinoma cells by the upregulation of Bcl-2-interacting mediator (Bim) expression [236]. Blask et al. [101] showed that there is a strong relation between phytomelatonin/melatonin contained in nutritional supplements and human cancer prevention. Another in vitro study suggested that melatonin is able to reduce liver cancer cell viability inhibiting the proliferation through the melatonin membrane receptor 1 (MT1). Moreover, a study revealed that melatonin by the downregulation the cyclooxygenase 2 (COX-2) expression and the Bcl-2/Bax ratio sensitizes human liver cancer cells to apoptosis induced by endoplasmic reticulum stress [237]. In vivo studies showed that melatonin protects from N-nitrosodiethylamine-induced liver tumour by reversing liver marker enzymes (ALT, AST), antioxidant levels and circadian clock disturbance. Furthermore, this indoleamine activates ER stress and inducing apoptosis [47,208].

## 7. Conclusions

**Figure 2:**
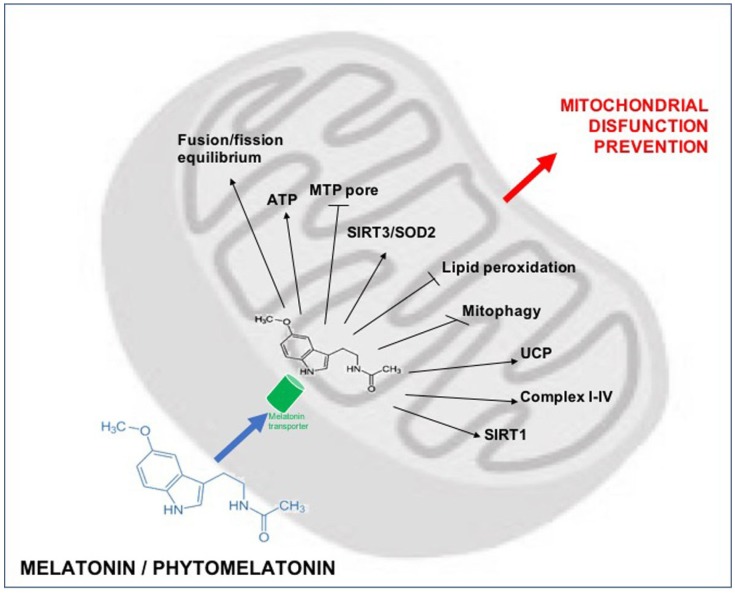
The targeting of melatonin to the mitochondria. Melatonin enters the organelles through specific oligopeptide transporters (PETP 1/2). In these organelles melatonin reduces ROS and prevent lipid peroxidation. Melatonin in mitochondria increase the efficiency of the electron transport chain and improve ATP production. ROS produced by this reaction are directly scavenged by melatonin but are also scavenged by mitochondria SIRT3 and metabolized by SOD2. Melatonin also modulates UCP2 for maintaining the inner mitochondrial membrane potential and prevents opening of the MPT pore. This limit the translocation of cytochrome c in cell cytosol and, so, prevents cellular apoptosis.

Data regarding the protective effects of food rich in melatonin/phytomelatonin in liver pathological conditions currently are not available. Nevertheless, the collected studies could suggest that the health-promoting effects ascribed to a dietary style with increasing of blood melatonin levels may be considered a promising preventive approach for a variety of liver diseases. In particular melatonin/phytomelatonin leads to the preservation of cellular homeostasis via downregulation of oxidative stress also at mitochondrial level (Figure 2). In these latter organelles melatonin/phytomelatonin enters through specific oligopeptide transporters (PETP 1/2) and performs its multiple functions. These actions, especially its antioxidant function, preserve mitochondrial function and benefit diseases in which mitochondrial malfunction is a feature, among which liver diseases.

An important take-home message from this review is that melatonin should not be thought of as a regular antioxidant in liver disease. The mere fact that it is both consumed in the diet and produced makes melatonin unique. Additionally, the fact that melatonin is associated with mitochondria should make it of significant interest in any study in which the endpoints include deferring the onset of diseases and improving the quality of life. However, it is important to report that the health benefits attributed to plant foods and Mediterranean or other diets could not depend on a single compound present in them (phenolic, carotenoid, etc.) but combination of other substances enhance the actions inducing synergic effects. Moreover, another limitation in the use of this indoleamine for targeting oxidative damage in liver is the fact that much of the results derived from studies on animal models and there are very few clinical studies. 

So, more research is needed to strengthen the potential beneficial effects of dietary melatonin/phytomelatonin in liver pathological conditions. 

## Figures and Tables

**Table 1:** Examples of melatonin content in plants, foods and drinks data from: [10,11,12].

Plant/Food/Drink	(Phyto)melatonin Content
Walnuts	3–4 ng/g
Strawberry	12 pg/g
Tomato	32 pg/g
Cereals	1000–1300 pg/g
Extra virgin olive oil	70–119 pg/mL
Wine	4000–5000 pg/mL
Beer	52–170 pg/mL
Apple	48 pg/g
Banana	0.66 ng/g
Pineapple	0.28 ng/g
Orange	0.15 ng/g
Green tea	250 ng/g
Chamomile	300 ng/g
Coffee	780 ng/mL
Chicken meat and skin	2.3 ± 0.23 ng/g
Chicken liver and heart	1.1 ± 0.01 ng
Lamb	1.6 ± 0.14 ng/g
Beef	2.1 ± 0.13 ng/g
Pork	2.5 ± 0.18 ng/g
Salmon	3.7 ± 0.21 ng/g
Solid dried eggs	6.1 ± 0.95 ng/g
Human milk	0–42 pg/mL
Cow milk	3–25 pg/mL
